# Desing and methodological process for assessing quasi-experiments in virtual reality environments for deepfake recognition in the artificial intelligence era

**DOI:** 10.1016/j.mex.2025.103242

**Published:** 2025-02-20

**Authors:** Alberto Sanchez-Acedo, Alejandro Carbonell-Alcocer, Manuel Gertrudix

**Affiliations:** Department of Audiovisual Communication and Advertising, University Rey Juan Carlos (ROR 01v5cv687), Camino del Molino 5, Fuenlabrada, Madrid 28942, Spain

**Keywords:** Methodological process, Quasi-experimental design, Quasi-experiment, Virtual reality, A-frame, Virtual environment, Deepfakes, Artificial intelligence, Disinformation, The challenges of media and information literacy in the artificial intelligence ecology: deepfakes and misinformation

## Abstract

Nowadays, the impact of artificial intelligence tools in the professional field must be analyzed, as well as their influence on the field of journalism and information. One of the aspects that has generated most concern in this area is the use of these tools, which can generate audiovisual content, for the creation of deepfakes. This article presents the methodology used to carry out a quasi-experiment designed to study and analyze the behaviour of young people in the face of possible exposure to deepfakes generated with artificial intelligence tools, as well as their ability to identify them. The experiment is conducted in a virtual environment in which participants are immersed in an interact with the environment in which they visualize newspaper front pages that include contextual elements. Participants must review the information included in the virtual environment to determine whether the images displayed correspond to real people or people generated with artificial intelligence tools. In addition, the influence and importance of the contextual elements accompanying an image in determining whether it is fake or real is analyzed. This article aims to detail the methodology used in this experiment to promote its replicability.

• This article proposes the method of a detailed guide to be replicated and reproduced in future academic research to understand the media diet of different population groups.
• Datasets are provided with results that allow for comparative, longitudinal and replication studies.
• The A-Frame framework for the design of virtual environments is introduced and can be used for the design of quasi-experiments.

## Graphical abstract

**Figure:**
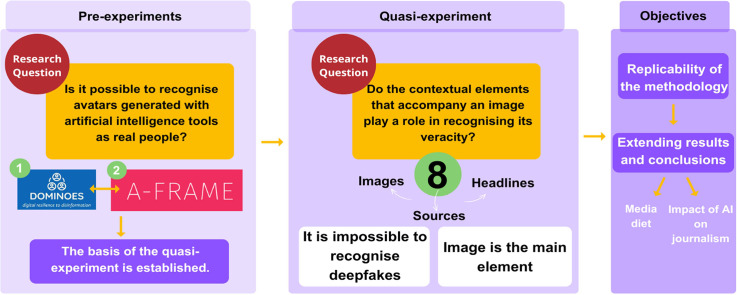

Specifications table

**Table:** 

Subject area:	Computer Science
More specific subject area:	*Virtual reality and artificial intelligence; communication and journalism*
Name of your method:	*The challenges of media and information literacy in the artificial intelligence ecology: deepfakes and misinformation*
Name and reference of original method:	*Sanchez-Acedo, A., Carbonell-Alcocer, A., Gertrudix, M. & Rubio-Tamayo, J.* L. *(2024a). Resultados preexperimento “Retos de la Alfabetización Mediática e Informacional en la ecología de la Inteligencia Artificial: deepfakes y desinformación”*. https://doi.org/10.5281/zenodo.11099500 *Sanchez-Acedo, A., Carbonell-Alcocer, A., Gertrudix, M. & Rubio-Tamayo, J.* L. *(2024b). Guía del cuasiexperimento “Retos de la Alfabetización Mediática e Informacional en la ecología de la Inteligencia Artificial: deepfakes y desinformación”.*https://doi.org/10.5281/zenodo.11094731 *Sanchez-Acedo, A., Carbonell-Alcocer, A., Gertrudix, M. & Rubio-Tamayo, J.* L. *(2024c). Resultados del cuasiexperimento “Retos de la Alfabetización Mediática e Informacional en la ecología de la Inteligencia Artificial: deepfakes y desinformación”.*https://doi.org/10.5281/zenodo.11093933 *Sanchez-Acedo, A., Carbonell-Alcocer, A., Gertrudix M. & Rubio-Tamayo, J.*L. *(2025). Códigos HTML de los ambientes virtuales para el experimento “Retos de la Alfabetización Mediática e Informacional en la ecología de la Inteligencia Artificial: deepfakes y desinformación”.*https://doi.org/10.5281/zenodo.14718070
Resource availability:	*Hardware:* * 1. Meta Oculus Quest 2 (*https://www.meta.com/es/quest/products/quest-2//*)* *Software:* *1. Synthesia (*https://www.synthesia.io/es*)* *2. Canva (*https://www.canva.com/*)* *3. A-frame (*https://aframe.io/*)* *4. Glitch (*https://glitch.com/*)* *5. Internet connection* *6. Access to the virtual environments of the pre-experiments:* *- First environment (*https://bit.ly/ifema23cw) *- Second environment (*https://bit.ly/ofilibre23cw) *7. Access to the virtual environments of the quasi-experiment:* *- Control group (*https://bit.ly/CONTROL2023) *- Experimental group (*https://bit.ly/EXPERIMENTAL2023) *The HTML code for points 6 and 7 is available at* Sanchez-Acedo et al. [23] *Data collection:* *1. Microsoft forms*

## Background

In the ecology of new realities, where extended technologies merge with artificial intelligence tools, a revolutionary new ecosystem emerges that enhances the quality of immersive experiences [9]. In this new revolution, artificial intelligence is the key player, and such transformational progress has been compared to the Industrial Revolution of 1760 [4]. Artificial intelligence tools have been introduced in different professional fields modifying the conception and execution of management, tasks and processes [16,26]. In the field of communication and journalism, artificial intelligence has, among other effects, the harmful capacity to misinform through the generation of deepfakes [8,14]. That is, multimedia material that has been manipulated and whose authenticity and veracity cannot be easily recognized [1]. The manipulation of journalistic images has existed since the last century, when in the First World War several photographers made visual compositions to generate greater impact on the viewer [11]. Today, artificial intelligence tools, capable of generating avatars and representing real people, carry with them the implicit threat of inducing possible bewilderment in the human mind [18]. This generates confusion in the spectators, which will make them doubt whether what they are seeing is false or true [7]. In consequence, the good practice and use of artificial intelligence must be regulated. Similarly, educational actions should be developed, from the earliest levels of education, aimed at raising awareness of possible exposure to something that is not real [3].

To study this phenomenon, we present the methodology used in a quasi-experiment that analyses the degree of recognition of deepfakes in virtual environments [18]. The study is framed in the posthumanist virtual world, whose principle lies in decentralizing the human being to the benefit of the new realities of artificial intelligences, which are integrated into the immersive spectrum where they come to life ([5]; Lorenz et al. [12]). Participants will be immersed in an artificially generated universe, where they can dissociate from the real world and explore its sensory limits [13], which leads them to new concepts never seen before. This fusion gives life to the quasi-experiment, which presents two virtual environments in which participants visualize recreations of newspaper front pages composed of a headline, the source of information and an image, which must be recognized as real or deepfake.

This article describes the methodological process developed for the conceptualization, design and execution of the quasi-experiment. It is described in detail from the initial phase with the previous pre-experiments that make it up, to its final phase. The methodology described here aims to be replicated in future research of this nature and to analyze the degree of recognition of deepfakes generated with artificial intelligence tools, in addition to being able to describe the media literacy of different population groups. In this way, the aim is to reach conclusions about the current way in which information is consumed in the new digital ecosystems.

## Method details

**Fig. 1:**
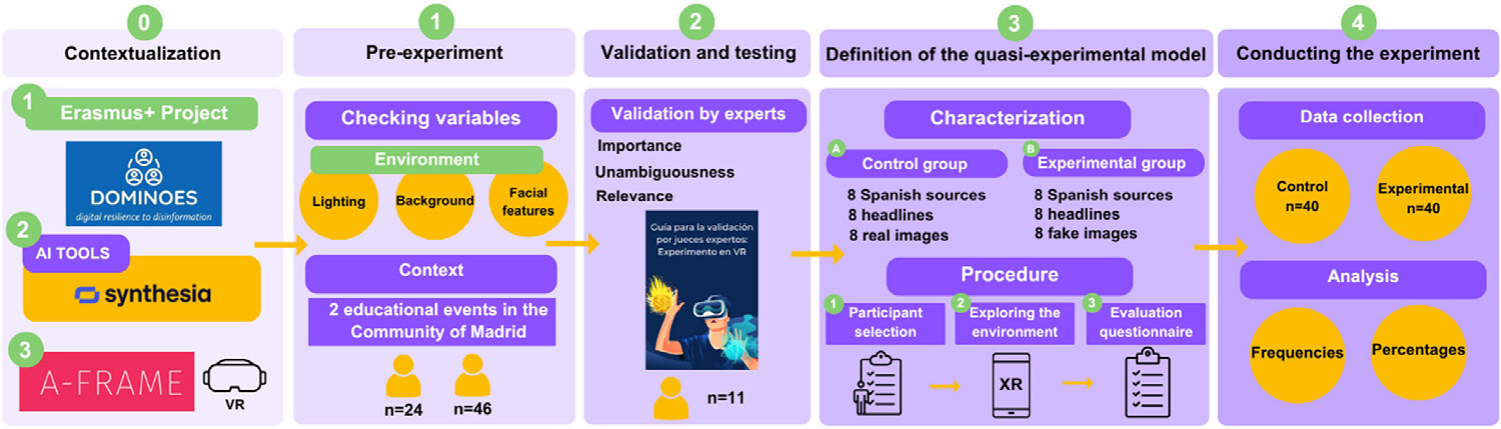
Methodological process carried out.

This research was proposed with the motivation to study the phenomenon of artificial intelligence and its impact on the world of information and journalism. Fig. 1 shows the entire methodological process followed, from the design of the pre-experiments to the final phase of collecting and analyzing the results of the quasi-experimental model.

**Step 0. Contextualization**

The study is part of the Erasmus+ DOMINOES project (Digital Competences INformatiOn EcoSystem 2021–1-RO01-KA220-HED-000,031,163) [6], which pursues the general objective of reducing social polarization and combating fake news and disinformation. Thus, it aims to detect and counter propaganda and manipulation of information. This research is presented as a tool to achieve the project's objectives. It sets out a complete methodology that indicates the guidelines for carrying out experiments to determine the degree of recognition of deepfakes in different population groups. In this way, it will be possible to establish mechanisms to combat misinformation.

**Fig. 2:**
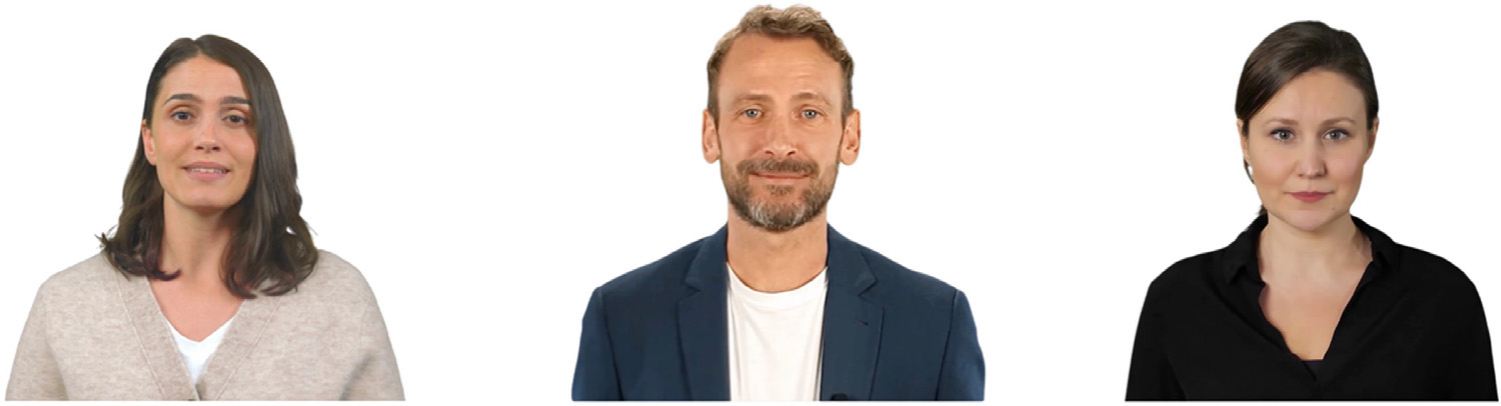
Images of avatars generated with synteshia.io.

Among the tasks carried out in the DOMINOES project, an evaluation of the different artificial intelligence tools used in the professional field of information is made. Among them, those capable of generating avatars in image and video format and which can be used as deepfakes are analyzed, as in the case of Synthesia.io [2]. It is precisely with this tool that all the materials that conform the whole experiment are worked on and elaborated from the beginning. Fig. 2 shows some of the avatars that the tool offers.

As can be seen in the image, Syntesia.io is able to generate videos and images of avatars that simulate real people. Thus, the initial research question of the experiment is established: Is it possible to recognize that these avatars have been generated with artificial intelligence tools, and are they mistaken for real people?

With these initial questions about the recognition of deepfakes, the first pre-experiments are planned.

On the other hand, environments designed with virtual reality tools are used that allow participants to be sensorially isolated. Thus, their main stimulus will be the virtual environment where they see the images. For the creation of these virtual spaces, both in the pre-experiments and in the quasi-experiment, the A-Frame tool is used. This tool allows to generate virtual spaces in a simple way that can be displayed on the web using HTML tags [10,15]. To start working with this tool, a guide is available in Sanchez-Acedo [22], in addition to consulting all the documentation available on their website. The design of the virtual spaces is designed so that participants can see the entire environment with the virtual reality glasses and without the need to move around, just by moving their heads. Finally, the HTML content is edited on the online platform Glitch.com, which provides a link to the scene that is then shortened with bit.ly and easily embedded in the VR headset. All HTML codes presented in this research are available in Sanchez-Acedo et al. [23].

**Step 1. Pre-experiment**

The aim of the pre-experiment is to explore, in an initial way, the degree of recognition of avatars generated with artificial intelligence tools, in this case with Synthesia.io, which can be used as deepfakes. To this end, two pre-experiments were carried out, the first one at the Madrid es Ciencia fair in Ifema, and the second one at the OfiLibre conference at the University Rey Juan Carlos, with 24 and 46 participants, respectively.

### Design of virtual environments and images

**Fig. 3:**
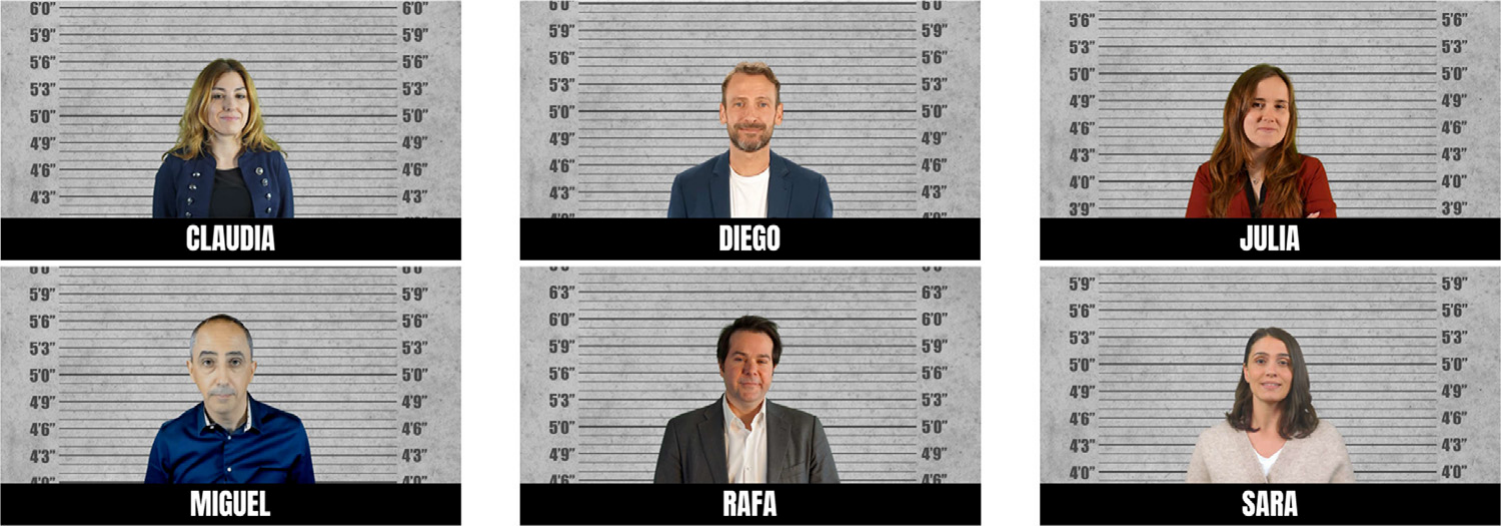
Images of the pre-experiment carried out at Madrid es Ciencia fair.

The virtual environments of the two pre-experiments were designed using A-Frame. In these, the images of the people are placed around the participant's initial point of view. A neutral background was established for the design of these images, simulating a figure wheel and mid-plane of the person. In addition, each person is given a fictitious name. The images used for the first of the pre-experiments are presented in Fig. 3.

In the environment of the first pre-experiment, carried out at the Madrid es Ciencia fair, 4 of the images are real people, members of the Ciberimaginario research group of University Rey Juan Carlos. In the case of Sara and Diego, they are generated with Synthesia.io, and in the case of Miguel, it is an avatar created from a real image.

**Fig. 4:**
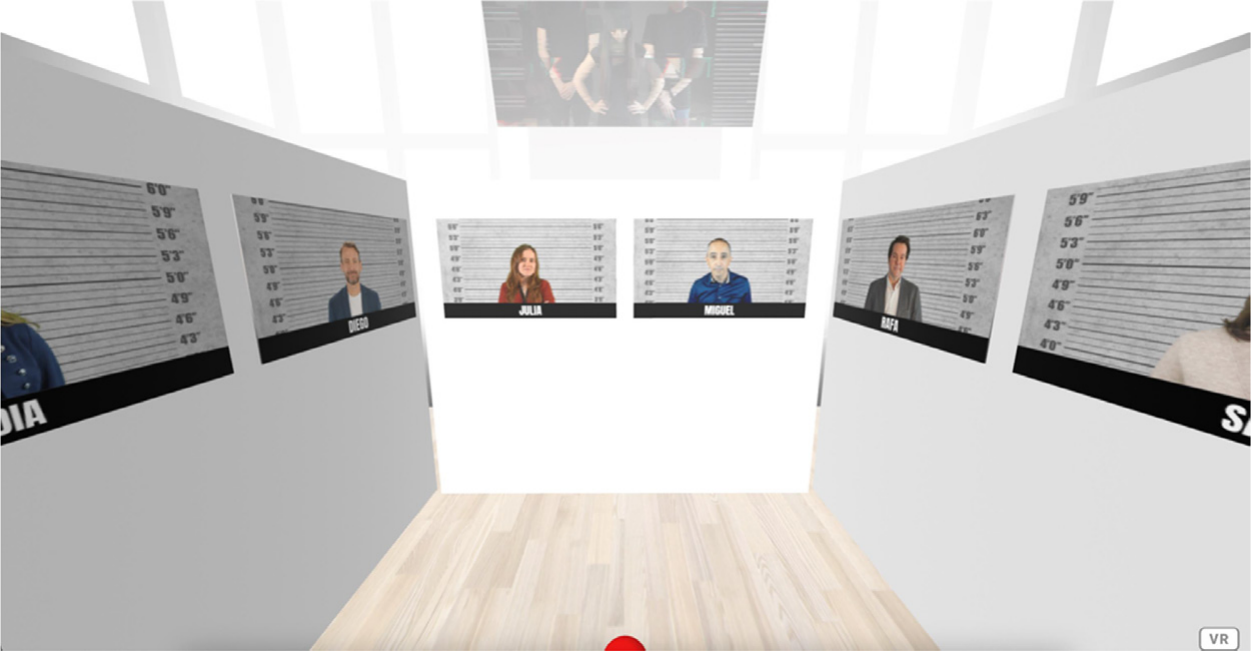
Virtual environment of the first pre-experiment. Available in: https://bit.ly/ifema23cw.

These images are integrated into the A-Frame environment presented for the first pre-experiment, as shown in Fig. 4.

**Fig. 5:**
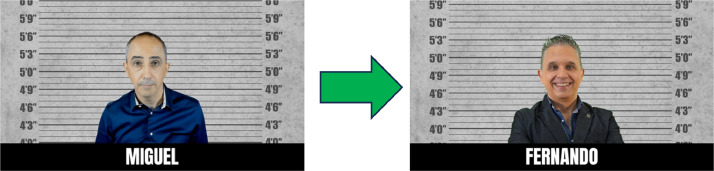
Image substitution for the second pre-experiment.

For the following pre-experiment, carried out at the OfiLibre conference of the University Rey Juan Carlos, it was decided to replace the image of Miguel with another image of a real person, also a member of Ciberimaginario, as shown in Fig. 5. In this way, the environment of the second pre-experiment has 4 images of real people and 2 generated with artificial intelligence.

**Fig. 6:**
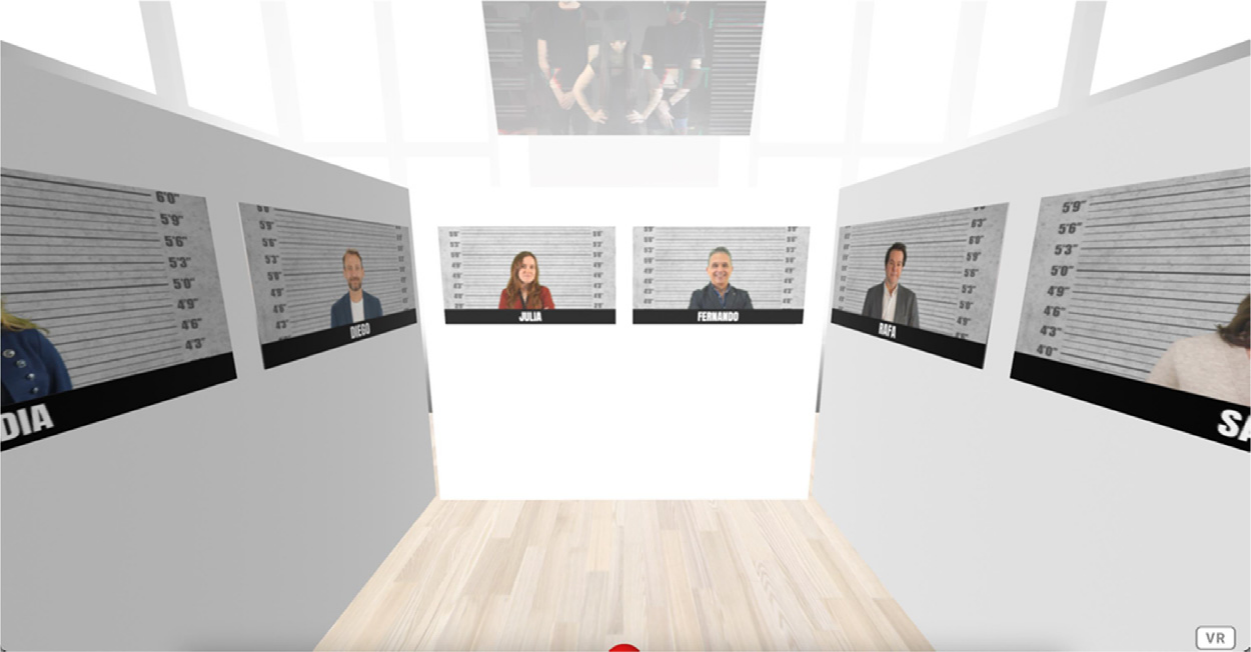
Virtual environment of the second pre-experiment. Available in: https://bit.ly/ofilibre23cw.

The A-Frame environment presented in the second pre-experiment is shown in Fig. 6.

### Dynamics of conducting the pre-experiments and results

**Fig. 7:**
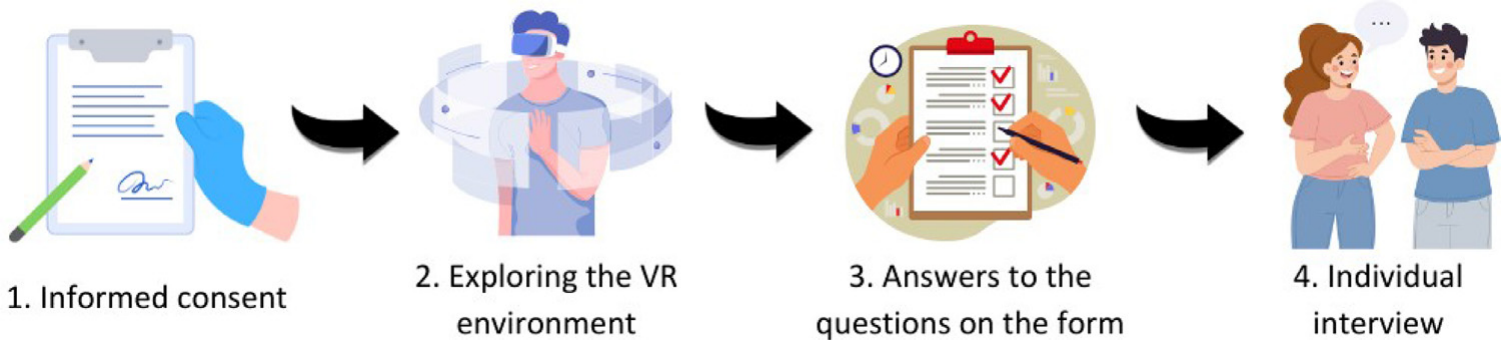
Pre-experiment steps.

The procedure for the pre-experiments consists of the participants exploring the environments designed for image identification with the virtual reality headset. Once they have done so, they must indicate, using a form created with Microsoft Forms, which of the images they believe to be real, and which are generated by artificial intelligence tools. Before starting the exploration, each of the participants must give their consent to participate by means of a form, the content of which is shown in the first section of Table 1. Once they have done so, the researcher will give them the dynamic instructions to start the experiment, in addition to providing the virtual reality headset with the environment previously loaded on the web. During the exploration, participants will be asked to examine the images closely so that they can recognize which are real and which are not. Once they have finished their immersive experience, they will take off the headset and the researcher will again provide them with the form for them to fill in their answers. Fig. 7 summarizes the dynamics of the pre-experiment.

**Table 1:** Model of the questions included in the pre-experiment form.

Ítem/Question	Content
Authorization Section	
I consent - Age	I declare that I am at least 16 years old
I consent - Aim of the study	I understand what this study is and what it aims to achieve
I consent - Procedure	I understand how the study will be conducted
I consent - Data processing	I understand how my data will be used
I consent - Data protection	I understand my data protection rights
I consent - Free participation	I declare that I freely participate in the study
I consent - Use of data	I consent to my data being processed for the purpose of this study

Sociodemographic variables	

Gender	Male/Female/Non-binary/Other
How old are you?	*Open question*

Image Recognition Section	

Sara	Real/AI
Miguel	Real/AI
Claudia	Real/AI
Diego	Real/AI
Julia	Real/AI
Rafa	Real/AI

Other	

If you wish to make any further contribution	*Open question*
Comments during the experiment	*Open question*

The questions for the development of the pre-experiment are shown in Table 1. The first section ‘Authorization’ explains how the data will be processed and all relevant information. Participants must read it carefully and once they agree, they must give their consent to each of the items in this section. Next, the demographic variables on gender and age of participants are collected. The next section collects the questions on image recognition. Finally, two questions are included open for comments and contributions from the participants.

Finally, when they have finished filling in the form, participants are asked what aspects have conditioned them to decide whether the images are real or AI-generated. As they answer, the researcher records the answers in a table as shown in Table 2. The aspects asked include the composition of the image, the colour, the lighting, the definition/sharpness of the image or the features of the person, as well as other elements that the participants considered relevant.

**Table 2:** Table to collect the participants' responses at the end of the pre-experiments and to collect the key aspects to determine the nature of the images.

	Composition	Colour	Lighting	Definition/Sharpness of the image	Features of the person	Other
PARTICIPANT 1						
PARTICIPANT 2						
PARTICIPANT						

After analyzing the results of these pre-experiments, it is concluded that it is impossible to distinguish between real people and people generated with artificial intelligence. The characteristics listed in Table 2 do not allow the identification of these images. Thus, most of the participants finally declared that they had randomly selected their answers on the veracity of the images.

**Fig. 8:**
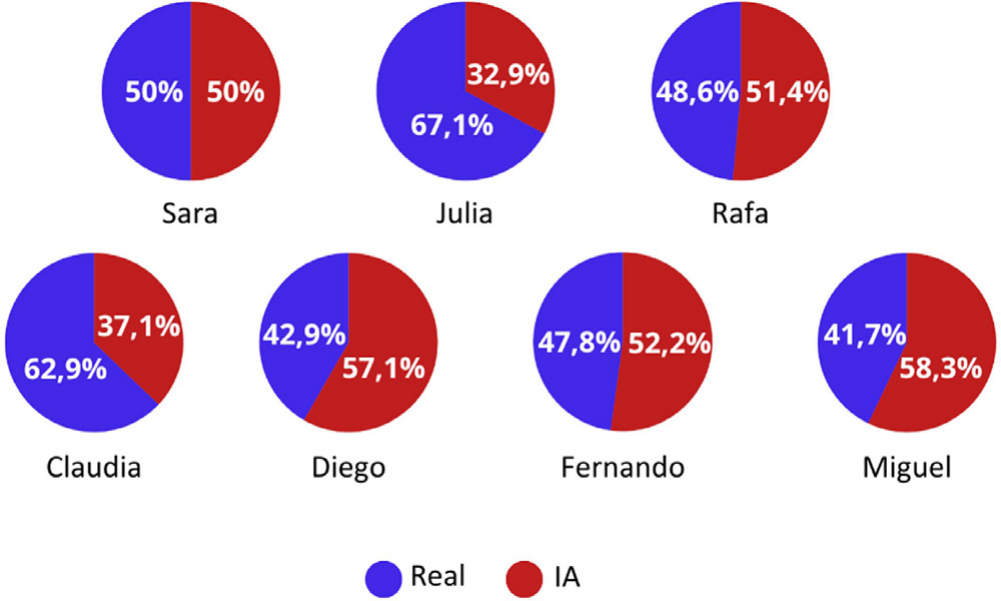
AI-generated/real person recognition relationship.

Fig. 8 shows the recognition ratio of real people and artificial intelligence for each of the images for the sum of the 70 subjects who participated in both pre-experiments.

These results allow us to conclude that there is no clear pattern to determine whether there are characteristics that indicate the veracity of the avatars. All these results can be found in Sanchez-Acedo et al. [19]. Thus, thanks to these results, it is possible to validate and establish the criteria for the quasi-experiment aimed at studying the current media diet of young people.

**Step 2. Validation and testing**

Before conducting the experiment, the form to be filled in by the participants is validated by judges who are experts in virtual reality, following criteria of relevance, unambiguousness and importance [24]. This guarantees the achievement of the objectives established and allows a correct analysis of the established variables of the object of study.

The expert judges must evaluate the importance, unambiguousness and relevance of each question proposed for the form following a Likert Scale. Similarly, the sociodemographic data of the expert judges are collected. Table 3 shows the questionnaire to be filled in for the validation of the experiment form.

**Table 3:** Validation questionnaire for expert judges.

Ítem	Content
Sociodemographic variables of expert judges	

Gender	-Male -Female -Other
Academic Level	-Bachelor's Degree -Master's degree -Doctorate
Expertise	*Open question*
Professional Position	*Open question*
Employment	*Open question*
Years of experience	*Open question*

Question validation section	

Question 1 is unambiguous	-Completely agree -Agree -Neither agree nor disagree -Disagree -Completely disagree
Question 1 is relevant	-Completely agree -Agree -Neither agree nor disagree -Disagree -Completely disagree
Question 1 is important	-Completely agree -Agree -Neither agree nor disagree -Disagree -Completely disagree
Comments to question 1	*Open question*
Question 2 is unambiguous	-Completely agree -Agree -Neither agree nor disagree -Disagree -Completely disagree
Question 2 is relevant	-Completely agree -Agree -Neither agree nor disagree -Disagree -Completely disagree
Question 2 is important	-Completely agree -Agree -Neither agree nor disagree -Disagree -Completely disagree
Comments to question 2	*Open question*
	…
General comments or contributions	*Open question*

The guide for validation and testing, as well as the responses of the expert judges, are available in Sanchez-Acedo et al. [19,20].

**Steps 3 and 4. Quasi-experiment**

The aim of the experiment is to find out and analyze whether the contextual elements that accompany an image determine its veracity or not. Replicating the design of the virtual environments of the pre-experiments, here the images will be recreations of newspaper front pages composed of an image of a person accompanied by a source of information and a headline. Thus, the initial research question is established: Does the contextual information (source and headline) accompanying an image influence whether it is real or AI-generated?

The experiment follows the quasi-experimental model that divides the participants into a control group and an experimental group [17], in which 80 young students from the Community of Madrid aged between 20 and 29 participated. The aim is also to analyze how young people access and consume information, as well as the real impact of artificial intelligence and deepfakes in the field of journalism.

### Design of the environment and recreation of the newspaper front pages

For each of the two groups a virtual environment is designed using A-Frame. The HTML codes of these environments, as well as those of the pre-experiment, can be found in Sanchez-Acedo et al. [23]. The design of the pre-experiment environments is replicated, where the images are placed around the participant's initial point of view. In this case, 8 newspaper front pages are recreated by designing a common background for all of them in which the three elements studied in this experiment are added: the image, the headline and the source of information.

**Fig. 9:**
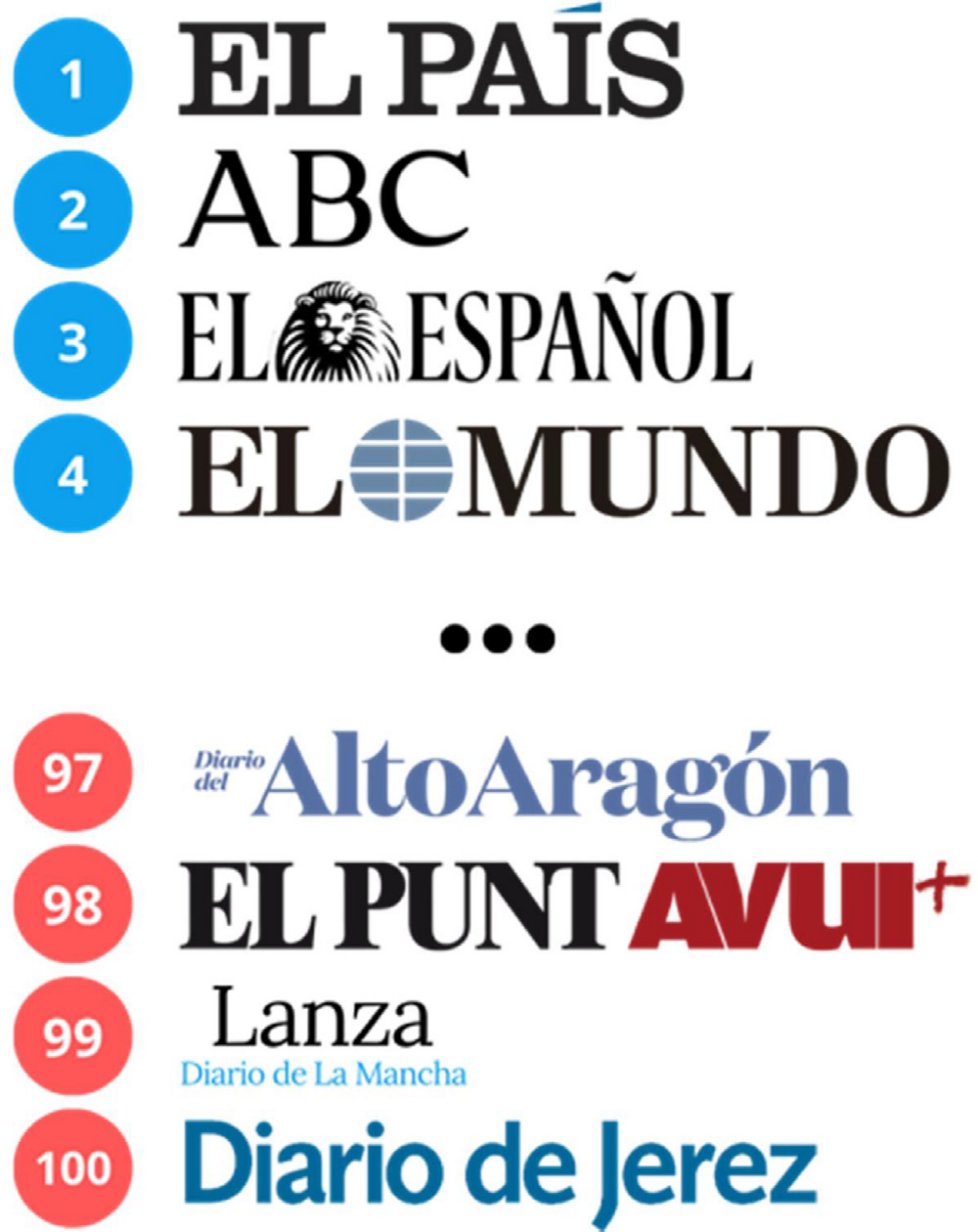
Media selected for the research in the ranking of the 100 most influential media at national level in Spain by the Global Ranking of Media Web Reputation of the SCImago Media Ranking.

To select the sources of information for each of the front pages, the Global Ranking of Media Web Reputation of the SCImago Media Ranking of the spring 2023 version [25] is used. Thus, the 4 most influential media at national level in Spain and the 4 least influential media among the list of the top 100 are selected, as shown in Fig. 9. This ranking compiles an annual and global list of the reputation of web media according to region, country or language.

To recreate the headline and the images on the front page, the selected media are searched for news items whose information is related to a real person. The photos of these people are then added to the front pages of these media.

**Fig. 10:**
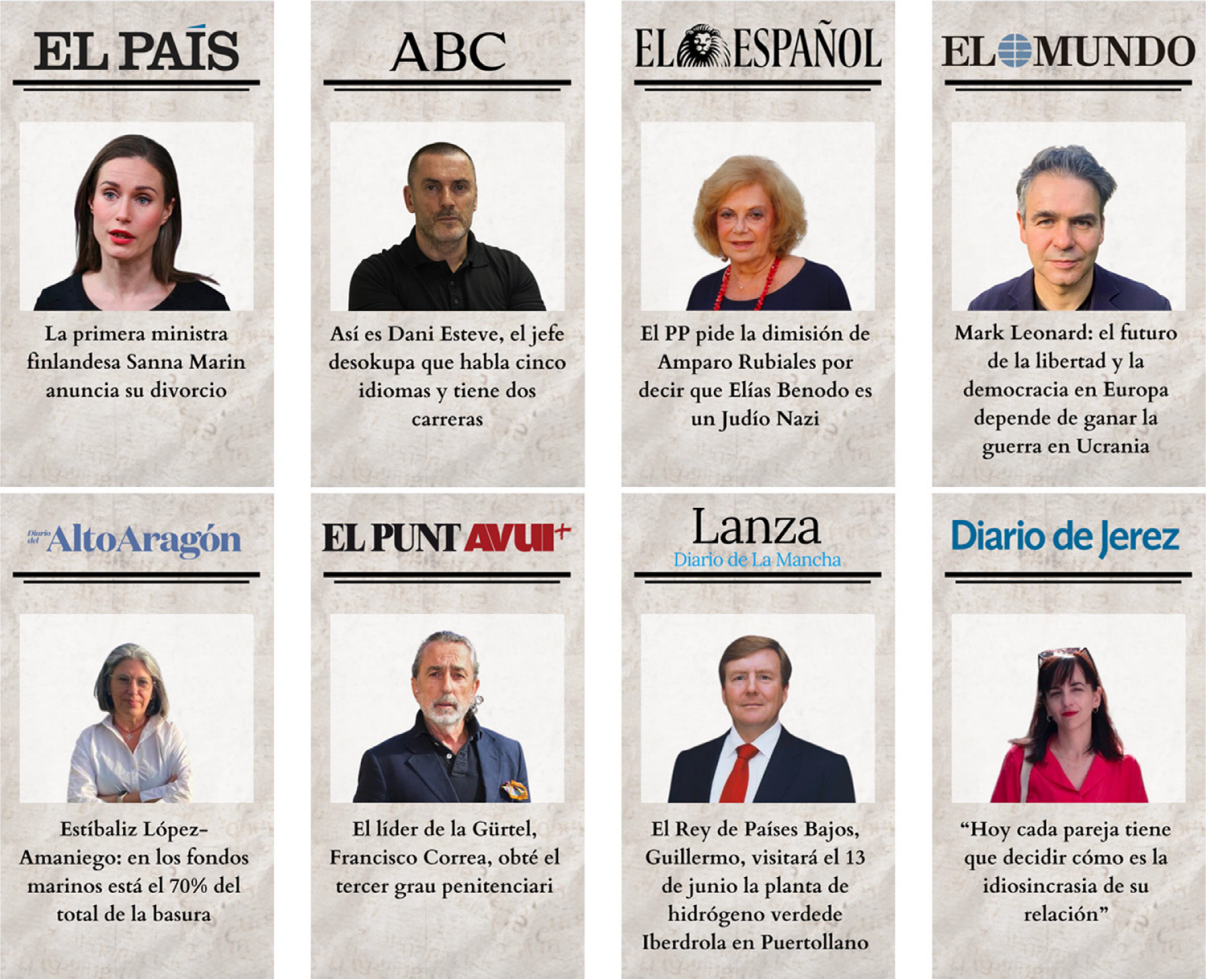
Recreations of newspaper front pages with real people included in the virtual environment of the control group.

In the virtual environment of the control group, all the images of people that appear are real. Fig. 10 shows the front pages of the control group, as well as the real headlines retrieved from their corresponding information source.

**Fig. 11:**
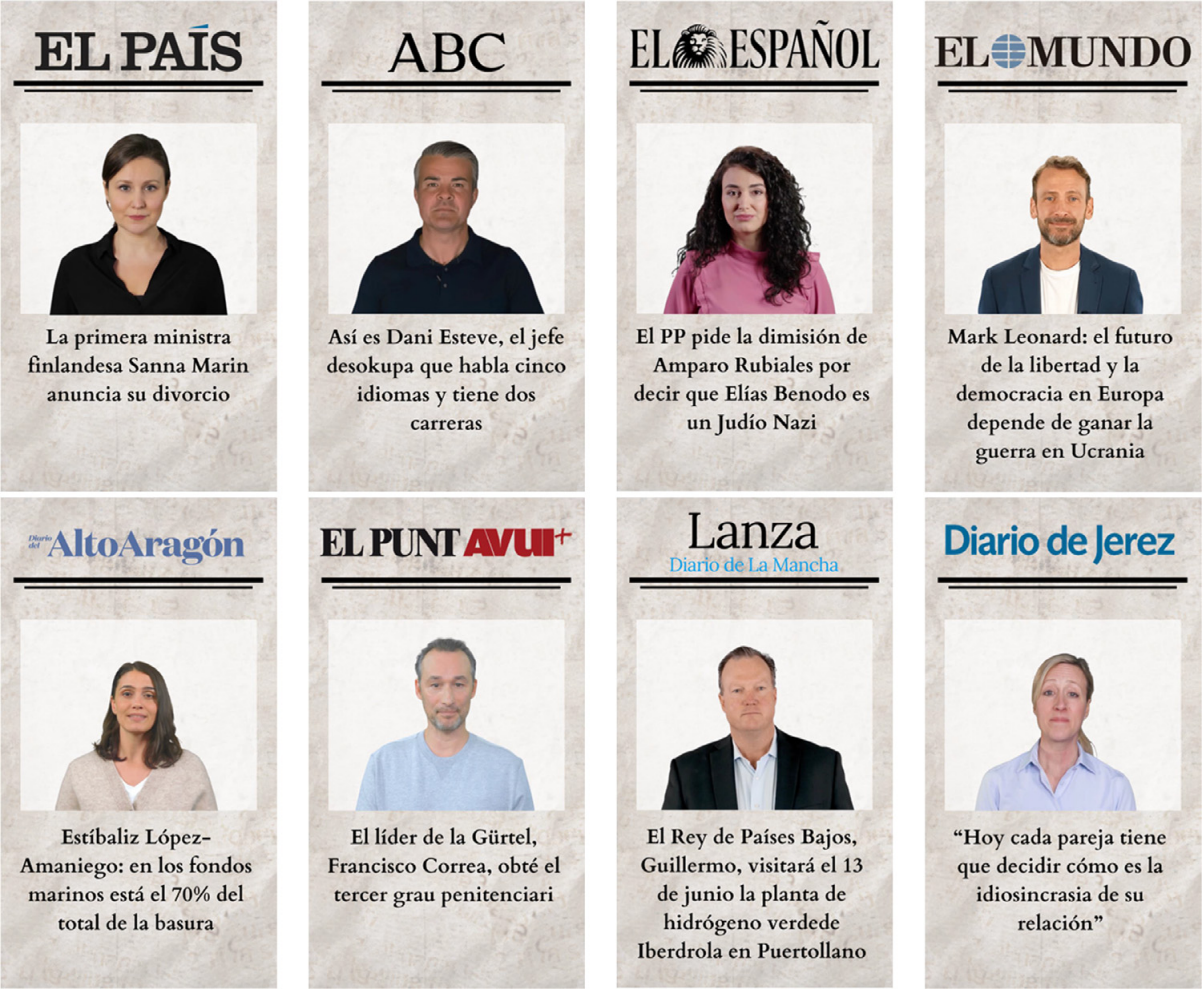
Recreations of newspaper front pages with AI-generated people included in the virtual environment of the experimental group.

In the case of the experimental group, the images of people are avatars generated with Synthesia.io. Similarly, the same media and real headlines were used as in the control group. Fig. 11 shows the newspaper front pages of the virtual environment of the experimental group.

**Fig. 12:**
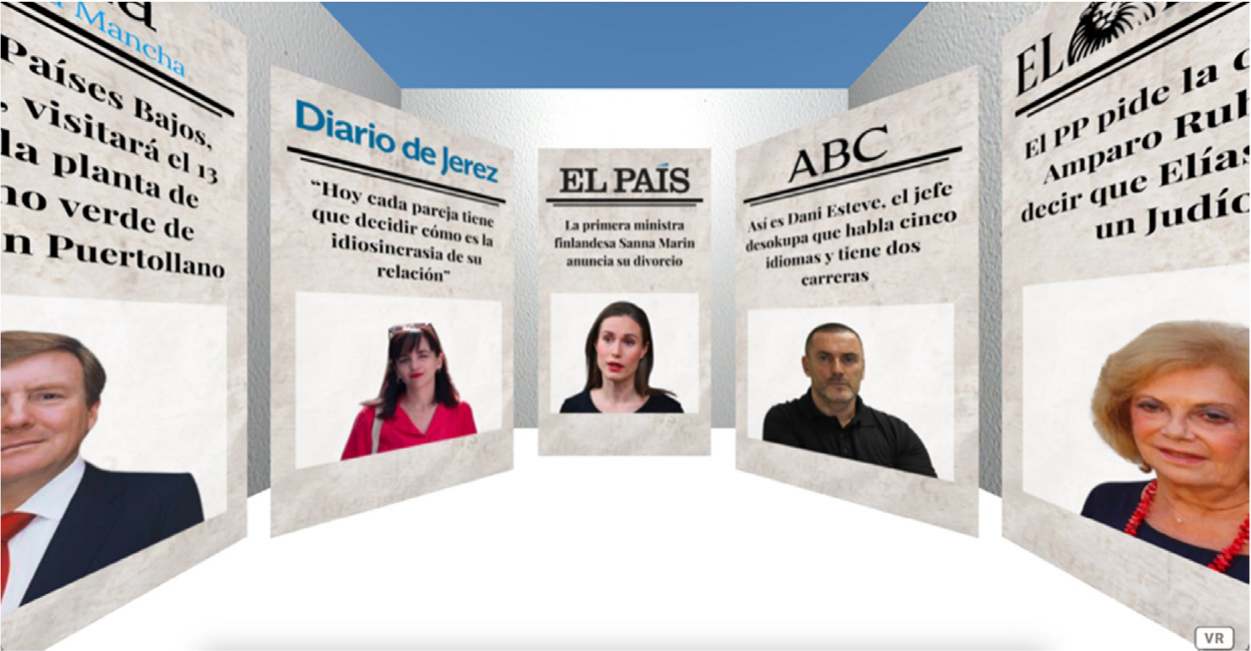
Virtual environment of the control group. Available in: https://bit.ly/CONTROL2023.

**Fig. 13:**
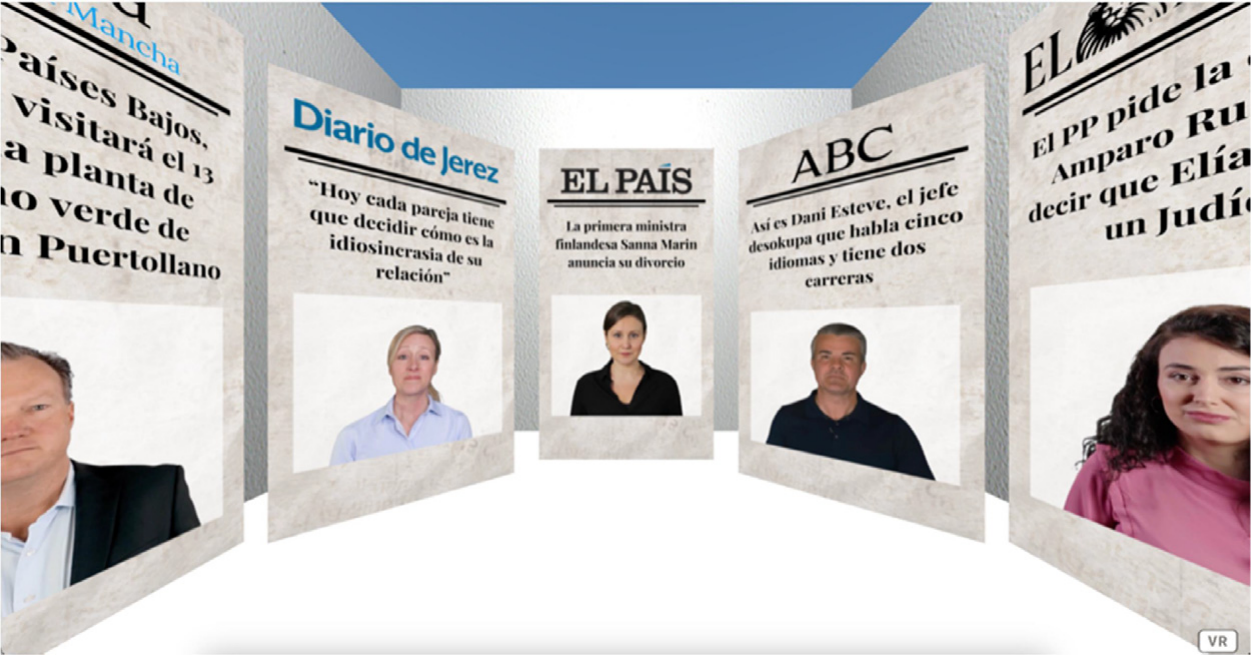
Virtual environment of the experimental group. Available in: https://bit.ly/EXPERIMENTAL2023.

For both groups, newspaper covers are placed around the participant's initial position in the virtual environments, as shown in Figs. 12 and 13.

### Dynamics of carrying out the quasi-experiment and form

**Fig. 14:**
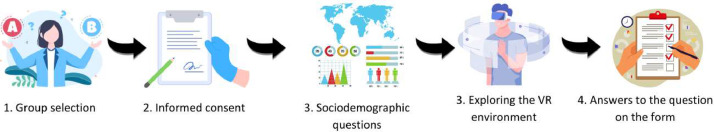
Dynamics of the quasi-experiment.

For the dynamics of the experiment, the steps are as shown in Fig. 14, which replicates the one used in the pre-experiments.

Once the participants have agreed to participate in the experiment, they will be randomly assigned to the control or experimental group. They then visualize the environment of with their groups using the virtual reality headset. At the end, they will answer the questions of the experiment as shown in the form presented in Table 4.

**Table 4:** Quasi-experiment form.

Ítem/Question	Content
Group selection section (to be filled in by the researcher)	
Group	Control group / Experimental group
Authorization Section	
I consent - Age	I declare that I am at least 16 years old
I consent - Aim of the study	I understand what this study is and what it aims to achieve
I consent - Procedure	I understand how the study will be conducted
I consent - Data processing	I understand how my data will be used
I consent - Data protection	I understand my data protection rights
I consent - Free participation	I declare that I freely participate in the study
I consent - Use of data	I consent to my data being processed for the purpose of this study
Sociodemographic variables	
Which gender do you identify with?	Male/Female/Other/I prefer not to say
How old are you?	*Open question*
Please, indicate your current education level	-Primary education -Compulsory Secondary Education -Basic Vocational Training -Intermediate Level Training Cycle -Higher Level Training Cycle -Baccalaureate -Bachelor's Degree -Master's Degree -Doctorate
Are you studying a degree at a university in Madrid?	Yes/No
If Yes Which university?	-Universidad de Alcalá -Universidad Autónoma de Madrid -Universidad Carlos III de Madrid -Universidad Politécnica de Madrid -Universidad Rey Juan Carlos -Universidad Complutense de Madrid
Please enter your postcode	*Open question*
Which of the following best describes the way you inform yourself?	-I don't consume informative content because it doesn't interest me. -I only review the headlines that are recommended to me, but I rarely access the content. -I review the headlines that are recommended to me, and I only skim the ones that most interest me. -I look for information that interests me in different information portals and I read it in depth.
Can you tell me the name of three places where you get information that you remember at the moment?	*Open question*
CONDUCTING THE EXPERIMENT	
Image Recognition Section	
Is the following subject real or generated by artificial intelligence? (Front page El País)	Real/AI
Is the following subject real or generated by artificial intelligence? (Front page ABC)	Real/AI
Is the following subject real or generated by artificial intelligence? (Front page El Español)	Real/AI
Is the following subject real or generated by artificial intelligence? (Front page El Mundo)	Real/AI
Is the following subject real or generated by artificial intelligence? (Front page Diario del Alto Aragón)	Real/AI
Is the following subject real or generated by artificial intelligence? (Front page	Real/AI
Is the following subject real or generated by artificial intelligence? (Front page Lanza Diario de la Mancha)	Real/AI
Is the following subject real or generated by artificial intelligence? (Front page Diario de Jerez)	Real/AI
Section importance of contextual elements	
Questions headline importance	
How important has the headline been in your opinion as to whether they are real or fake? (Front page El País)	-Not very important -Not important -Somewhat important -Important -Very important
How important has the headline been in your opinion as to whether they are real or fake? (Front page ABC)	-Not very important -Not important -Somewhat important -Important -Very important
How important has the headline been in your opinion as to whether they are real or fake? (Front page El Español)	-Not very important -Not important -Somewhat important -Important -Very important
How important has the headline been in your opinion as to whether they are real or fake? (Front page El Mundo)	-Not very important -Not important -Somewhat important -Important -Very important
How important has the headline been in your opinion as to whether they are real or fake? (Front page Diario del Alto Aragón)	-Not very important -Not important -Somewhat important -Important -Very important
How important has the headline been in your opinion as to whether they are real or fake? (Front page El Punt Avui)	-Not very important -Not important -Somewhat important -Important -Very important
How important has the headline been in your opinion as to whether they are real or fake? (Front page Lanza Diario de la Mancha)	-Not very important -Not important -Somewhat important -Important -Very important
How important has the headline been in your opinion as to whether they are real or fake? (Front page Diario de Jerez)	-Not very important -Not important -Somewhat important -Important -Very important
Questions image importance	
How important has the image been in your opinion as to whether they are real or fake? (Front page El País)	-Not very important -Not important -Somewhat important -Important -Very important
How important has the image been in your opinion as to whether they are real or fake? (Front page ABC)	-Not very important -Not important -Somewhat important -Important -Very important
How important has the image been in your opinion as to whether they are real or fake? (Front page El Español)	-Not very important -Not important -Somewhat important -Important -Very important
How important has the image been in your opinion as to whether they are real or fake? (Front page El Mundo)	-Not very important -Not important -Somewhat important -Important -Very important
How important has the image been in your opinion as to whether they are real or fake? (Front page Diario del Alto Aragón)	-Not very important -Not important -Somewhat important -Important -Very important
How important has the image been in your opinion as to whether they are real or fake? (Front page El Punt Avui)	-Not very important -Not important -Somewhat important -Important -Very important
How important has the image been in your opinion as to whether they are real or fake? (Front page Lanza Diario de la Mancha)	-Not very important -Not important -Somewhat important -Important -Very important
How important has the image been in your opinion as to whether they are real or fake? (Front page Diario de Jerez)	-Not very important -Not important -Somewhat important -Important -Very important
Questions source importance	
How important has the newspaper been in your opinion as to whether they are real or fake? (Front page El País)	-Not very important -Not important -Somewhat important -Important -Very important
How important has the newspaper been in your opinion as to whether they are real or fake? (Front page ABC)	-Not very important -Not important -Somewhat important -Important -Very important
How important has the newspaper been in your opinion as to whether they are real or fake? (Front page El Español)	-Not very important -Not important -Somewhat important -Important -Very important
How important has the newspaper been in your opinion as to whether they are real or fake? (Front page El Mundo)	-Not very important -Not important -Somewhat important -Important -Very important
How important has the newspaper been in your opinion as to whether they are real or fake? (Front page Diario del Alto Aragón)	-Not very important -Not important -Somewhat important -Important -Very important
How important has the newspaper been in your opinion as to whether they are real or fake? (Front page El Punt Avui)	-Not very important -Not important -Somewhat important -Important -Very important
How important has the newspaper been in your opinion as to whether they are real or fake? (Front page Lanza Diario de la Mancha)	-Not very important -Not important -Somewhat important -Important -Very important
How important has the newspaper been in your opinion as to whether they are real or fake? (Front page Diario de Jerez)	-Not very important -Not important -Somewhat important -Important -Very important

As with the pre-experiment form, another questionnaire was designed using Microsoft Forms in which the first section presents all the necessary information on the processing and use of the data of the participants, who must read it and give their authorization for each of the items in this section. In the next section, the sociodemographic data is collected, where the participants' level of studies and the way they access the information will be known. Then, the experiment is carried out by using the virtual reality headset. Once they have completed the visualization of the environment, they return to the form to answer the questions corresponding to the section on image recognition and the section on the importance of contextual elements. It is important to mention that, depending on the group to which the participant has been assigned, in these last two sections, the newspaper front pages of the control or experimental group appear, depending on the case of each participant. The results of the quasi-experiment are available in Sanchez-Acedo et al. [21].

### Technical considerations on the use of Meta Quest 2 VR headset

The experiments presented were carried out with the Meta Quest 2 virtual reality headset. This model is a practical option for the visualization of virtual environments thanks to its usability. This device stands out for its simplicity of use, as it has an interface similar to that of a smartphone mobile device.

The use of A-Frame makes it possible to develop displayable environments on the web, which allows, in a simple way, to visualize these environments directly from the browser application of the virtual reality headset device by activating the immersive experience option.

To configure the headset, it is necessary to delimit the safe play area in which the experience will take place. A guardian system is set up for this purpose. The participant leaves this play area, they will lose the view of the experience and will have to return to the delimited area.

Similarly, the headset must be used indoors, as the device has internal cameras that scan the physical space and must find walls or corners in order to establish a safe guardian system, which means that this cannot be done outdoors.

Finally, participants should be warned that the use of virtual reality headset can cause dizziness and other symptoms.

## Method validation

This article presents the methodology used to conduct this experiment for the first time. For its replicability, the entire process of conceptualization, design and execution of the experiment is detailed, and all the materials necessary for its reproduction are provided. These materials provided are excel files and datasets on the results of the experiment contextualized in Spain and adapted to the characteristics of the information context of the region. Thus, it is intended that these results can be compared with those of future research, which is a common objective in scientific research to validate and extend findings.

Replicating this methodology will allow new research to study the form of access to information in other population groups and compare results. In addition to analyzing the importance of the contextual elements that accompany an image when it comes to recognizing what is real and what is not. All of this will allow new conclusions to be drawn about the main aspects of the media diets of each group.

## Limitations

In order to replicate this research, certain limitations must be taken into account. The first of these is that the virtual environments must be adapted to the media context of the country or region where the experiment is being conducted. For this reason, rankings such as the SCImago Media Ranking or rankings specific to the area in which the study is carried out must be used.

On the other hand, the technical needs for the implementation and replicability of the experiment must be considered. Likewise, the use of extended reality devices is necessary to carry out the study, as well as technical knowledge in the creation and customisation of virtual environments, as in the case of A-Frame.

## Ethics statements

The Ethics Committee of the Rey Juan Carlos University has authorized the conduct of this experiment (Authorization ID 3,105,202,214,722), which involves the participation of human subjects. All the necessary procedures for its performance are approved, in accordance with the ethical standards and the requirements established in the protocol, in relation to the objectives of the study. In addition, the possible risks for the participants are justified. Likewise, the investigator's capacity and the means available are considered favourable and appropriate for carrying out the study.

The data obtained for the analysis of the results have been anonymized, guaranteeing the confidentiality of all participants.

## CRediT authorship contribution statement

**Alberto Sanchez-Acedo:** Conceptualization, Methodology, Investigation, Data curation, Writing – original draft. **Alejandro Carbonell-Alcocer:** Methodology, Investigation, Data curation, Writing – original draft. **Manuel Gertrudix:** Conceptualization, Methodology, Supervision, Writing – review & editing, Project administration, Funding acquisition.

## Declaration of competing interest

The authors declare that they have no known competing financial interests or personal relationships that could have appeared to influence the work reported in this paper.

## Data Availability

All the data are available on Zenodo repository. All links for the data are included in the manuscript.
